# Large-scale serological survey on *Mycobacterium avium* subsp. *paratuberculosis* infection in sheep and goat herds in Sicily, Southern Italy

**DOI:** 10.3389/fvets.2024.1334036

**Published:** 2024-02-01

**Authors:** Vincenzo Di Marco Lo Presti, Dorotea Ippolito, Sergio Migliore, Marco Tolone, Sebastian Alessandro Mignacca, Anna Maria Fausta Marino, Benedetta Amato, Rosita Calogero, Maria Vitale, Domenico Vicari, Flavia Pruiti Ciarello, Michele Fiasconaro

**Affiliations:** ^1^Istituto Zooprofilattico Sperimentale della Sicilia “A. Mirri”, Palermo, Italy; ^2^Unit of Emerging Zoonoses, Department of Food Safety, Nutrition and Veterinary Public Health, Istituto Superiore di Sanità, Rome, Italy; ^3^Department of Chemical, Biological, Pharmaceutical, and Environmental Sciences, University of Messina, Messina, Italy; ^4^Department of Agriculture, Food and the Marine-Pathology Division, Dublin, Ireland; ^5^Department of Veterinary Pathology, Bristol Veterinary School, University of Bristol, Bristol, United Kingdom

**Keywords:** paratuberculosis, seroprevalence, sheep, goat, *Mycobacterium avium* subsp. *paratuberculosis*, Italy, Sicily

## Abstract

**Introduction:**

Paratuberculosis (PTB) is a worldwide chronic, contagious enteric disease caused by *Mycobacterium avium* subsp. *paratuberculosis* (MAP) mainly affecting ruminant species. PTB is a WOAH-listed disease with direct and indirect economic losses in the livestock sector, negative impact on animal welfare and significant public health concerns. In spite of this, MAP prevalence in small ruminants is still unknown and the prevalence appears to be underestimated in many countries. The aim of this study is providing a first large-scale serological survey on MAP infection in small ruminants in Sicily, a region of Southern Italy with the 11.3 and 8.9% Italian national heritage of sheep and goats, respectively.

**Methods:**

For this purpose, we analyzed a total of 48,643 animals reared in 439 flocks throughout Sicily. MAP seroprevalence was estimated both at herd-level and animal-level within breeds reared in all the nine sampled provinces.

**Results:**

Our results revealed a high overall apparent prevalence at herd-level of 71.8% in sheep and 60.8% in goat farms with an animal-level prevalence of 4.5 and 5.1% in sheep and goats, respectively. Significant statistical differences were found between the provinces and within the breeds both in sheep and goats.

**Discussion:**

Our study provides the first large-scale serological survey on PTB infection in small ruminants in Sicily and showed a high prevalence of disease depending to the species, breed and province. This study represents the first step to better understand the MAP epidemiology in a typical Mediterranean breeding context, suggesting the need of in-depth study on the herds risk factors, including the eventual presence of candidate genes for resistance/susceptibility to PTB in native breeds.

## 1 Introduction

Paratuberculosis (PTB) or Johne’s disease is a chronic, contagious infectious disease that affects the enteric tract of domestic and wild ruminants. The causative agent of PTB is *Mycobacterium avium* subspecies *paratuberculosis* (MAP), which is mainly transmitted by the fecal-oral route and then spreads horizontally and vertically (1). The disease has been known and studied for over two centuries and it still represents a significant concern, both for the serious zoo-economic losses in infected herds and for its zoonotic potential (2).

Despite the lack of certainty on the cause/effect relationship, MAP is believed to be implicated in the pathogenesis of Crohn’s disease in humans, in consideration of the high probability of isolation of MAP in affected patients (3, 4). Furthermore, it is suspected that MAP may also be implicated in the pathogenesis of other diseases such as type 1 diabetes mellitus (5), multiple sclerosis (6), Parkinson’s disease (7), Blau syndrome (8), Hashimoto’s thyroiditis (9), and in other autoimmune diseases, although no firm evidence has been scientifically confirmed.

The main routes of infection of MAP in humans are represented by environmental contamination, drinking water (10) and the food chain (11). Milk and dairy products have been implicated in the animal-to-human transmission, both if consumed raw or pasteurized (12). Indeed, MAP is characterized by a high resistance in the environment and can survive the standard commercial pasteurization (11).

The confirmation of MAP as a zoonotic agent would make mandatory the implementation of control and eradication procedures, which would inevitably lead to major issues in terms of public and veterinary health and severe economic losses for the entire livestock sector.

Given the sanitary and economic relevance of PTB, information on the occurrence and prevalence of MAP are available from many countries but limited to cattle dairy herds, in which control plans are also in place. In contrast, few studies in small ruminants have been published worldwide and consequently, prevention and control programs have not been established in many countries. Both seroprevalence and risk factors study are needed, especially in semi-extensive and extensive breeding contexts, in order to plan adequate strategies for the control and eradication of disease. In ruminants, PTB is clinically evident during the later stage of the infection. Symptoms such as significant weight loss, emaciation, spontaneous death may be only apparent in advanced stages (13). Subclinically infected animals can eliminate MAP through faeces even during the early phase of the infection, making difficult the control of the disease (13, 14). Goats appear to be more susceptible than sheep and both species are likely to develop the clinical signs of the disease (15).

PTB is globally widespread. It has been reported in several countries, such as Italy (16), Germany (17) and France (18), as well as in Asia, Africa and Oceania (19, 20). Caprine PTB was reported in Canada (21), USA (22) and Brazil (23). In New Zealand, the disease is endemic and widespread in sheep and dairy goats (24). In the Middle East and Africa, PTB was reported in sheep and goats in Saudi Arabia (25), Jordan (26), Egypt (27), Sudan (28), Morocco (29) and South Africa (19).

Italy is one of the largest European countries in terms of number of sheep and goat herds with an estimated population of around 5,9 million sheep and around 1 million goats (Ministero della Salute, Sistema Informativo Veterinario – Statistiche. https://www.vetinfo.it/j6_statistiche/#/report-pbi/89, accessed on June 30, 2023) and Sicily is the second Italian region for the number of sheep (662,305 heads, 11.3% of the national heritage) and third, after Sardinia and Calabria, for goats (90,926 heads, 8.9% of the national heritage). Sheep and goat breeding in Sicily is mainly semi-extensive, characterized by pastures, sometimes shared with other flocks, and a supplementary diet especially in the dry seasons. The island’s biodiversity is enriched by a large number of autochthonous breeds (4 sheep and 5 goats) which constitute an invaluable heritage as a source of high-quality milk requested for typical dairy products (30). Genetic susceptibility to MAP infections in several small ruminant breeds has been investigated using quantitative and/or molecular genetics and despite low hereditability, all studies confirm genetic influence on paratuberculosis susceptibility (31). However, further genomic explorative studies to identify candidate genes and evaluate their prevalence are needed in small ruminants, especially in native breeds.

The aim of our study is to conduct the first large-scale investigation of MAP seroprevalence in small ruminants throughout Sicily providing the basis for further studies on risk factors analysis and genetic susceptibility in native breeds.

## 2 Materials and methods

### 2.1 Ethical statement

This study did not involve controls under EU Directive 2010 (2010/63/EU) and blood collection was not required with the benefit of animal welfare. The large-scale study on MAP seroprevalence in small ruminant herds in Sicily was carried out on sera samples collected by official regional veterinary services during the annual brucellosis monitoring plans in force in Sicily.

### 2.2 Study area

**Figure 1:**
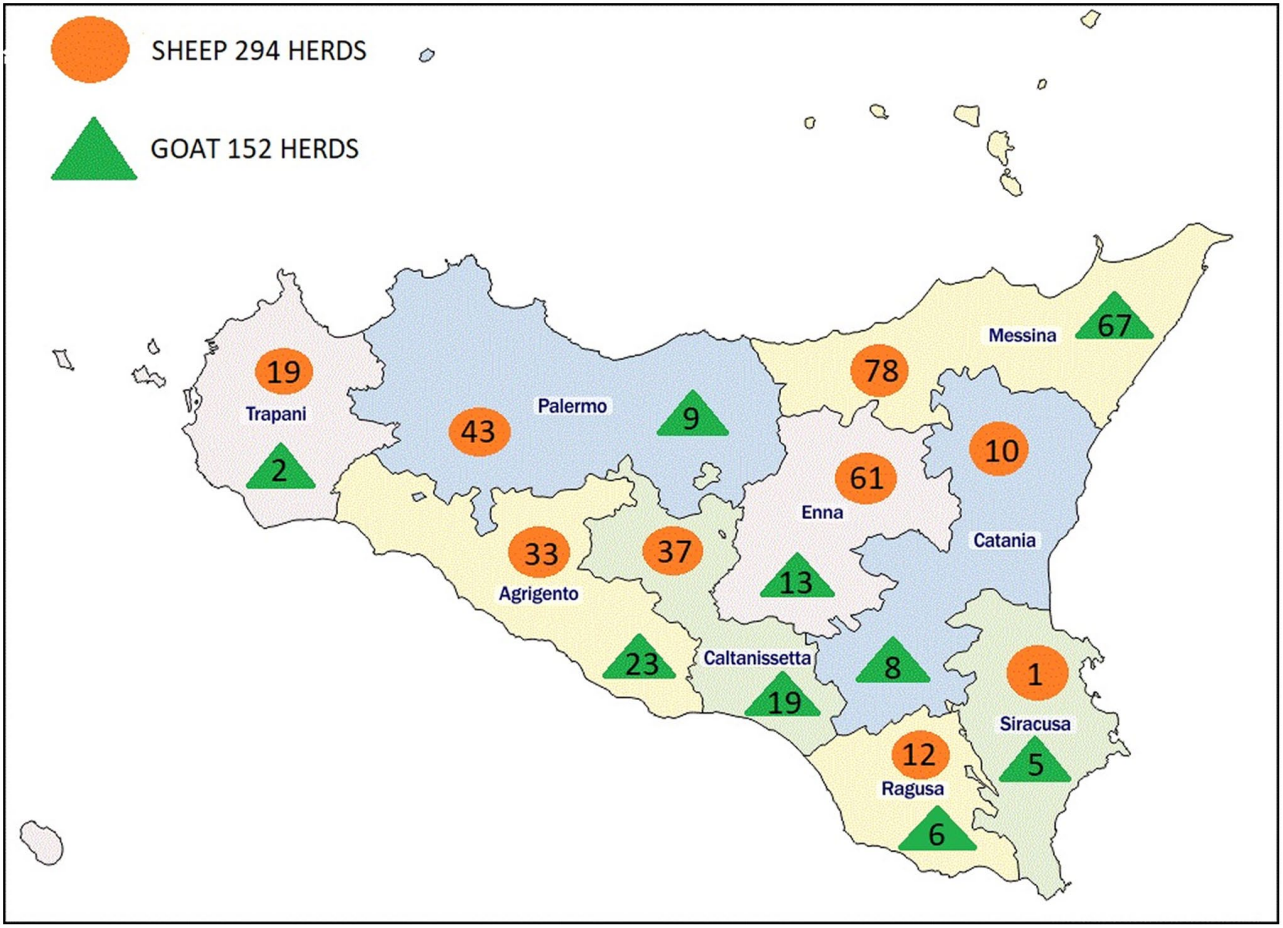
Map of the study area with sampled sheep and goat farms for province.

The study was carried out throughout the regional territory of Sicily, Southern-Italy, the largest island in the Mediterranean. It covers an area of 25,707 km2, including minor islands, and it is divided into nine provinces: Palermo, Trapani and Agrigento in the west, Caltanissetta and Enna in the center and Ragusa, Siracusa, Catania and Messina in the east (Figure 1).

### 2.3 Study animals

The analyzed samples belonged to dairy sheep and goats raised mainly in a semi-extensive system. Serum samples were obtained from 48,643 small ruminants, comprising 35,663 sheep and 12,980 goats. Regarding the sheep, we analyzed samples from crossbreed (18,848) and three different Sicilian native breeds: Comisana (3,914), Pinzirita (2,560) and Valle del Belìce (8,210). In goats we analyzed samples from crossbreed (7,746) and five different Sicilian native breeds: Derivata di Siria (453), Girgentana (324), Maltese (645), Messinese (1,088) and Argentata dell’Etna (7,746).

### 2.4 Study design and sampling method

Based on a previous study on the MAP seroprevalence carried out in sheep and goat in Sicily (Guercio et al., personal communication), we considered an estimated prevalence of 18% at herd level. Regarding the estimated prevalence at the animal level (32), reported a prevalence of 2%, obtained by Agar Gel Immunodiffusion Assay (AGID) method. However, given the low sensitivity in the preclinical phase of AGID method to detect MAP antibodies (26.8%) (33), the estimated prevalence at animal level was set at 5%.

Herds were selected by simple random sampling from official flock registers of Regional Government of Sicily. Vaccination plans against MAP have never been carried out in all sampled herds.

### 2.5 Sample size determination

The minimum sample size was calculated considering the total number of herds in Sicily (8,504 ovine herds and 3,121 caprine herds), using WinEpi software (http://www.winepi.net/, accessed on February 6, 2023) with 5% of precision and 95% confidence level (CI). Sampling was stratified by province based on the proportion of sheep and goats in each province. The stratified sampling was applied to calculate the minimum sample size at animal level, considering the number of heads reared in each farm by dividing the herds in six size class (from <50 to >1,000 animals). The total number of sheep or goat for each size class herds was calculated using WinEpi software (http://www.winepi.net/, accessed on February 6, 2023) with 5% of precision and 95% CI. In addition, seroprevalence was calculated within sheep and goat breeds at herd and animal level limited to the herds in which this information was available: 290 sheep herds (34,825 animals) and 149 goat herds (12,033 animals).

### 2.6 Serological analysis

Following serological testing for brucellosis, sera were collected, archived and stored at −18°C awaiting the analyses by the serology laboratory of Istituto Zooprofilattico Sperimentale della Sicilia “A. Mirri,” during the 3 years in study. All samples were examined by IDEXX Paratuberculosis Screening Ab Test [IDEXX (IDEXX Laboratories, Inc., Westbrook, ME, United States)] according to the manufacturer’s instructions. Positive samples were confirmed using IDEXX paratuberculosis verification Ab test (IDEXX Laboratories, Inc., Westbrook, ME, United States), following manufacturer’s instructions.

For calculating the true prevalence (TP), we used the Sensitivity (Se) and Specifity (Sp) data indicated by the producer: Se = 34.9% and Sp = 97.3% in sheep and Se = 51% and Sp = 94.8% in goats. Considering the low sensitivity of the test, we calculated the TP according to Se and Sp indicated in a review of 2009 by Nielsen & Toft (Se = 37% Sp = 98.5% in sheep and Se = 73% and Sp 97.5% in goats).

The TP was calculated using the Epitools calculation system which applies the corrective formula of Rogan-Gladen (34).

Since both Se and Sp at herd level are a function of sample size (*n*), and as the sample sizes per herd in this study varied considerably, the TP was estimated only at animal-level whereas apparent prevalence (AP) was used also at herd-level.

### 2.7 Statistical analysis

As previously mentioned, the Epitools software was utilized to calculate the AP and TP. The same software was employed to determine the confidence intervals for both AP and TP estimations, consistent with Brown et al. (35), along with the positive predictive value (PPV) and negative predictive value (NPV). The chi-square test was used to assess provincial and breed-within-species differences in apparent prevalence using the chi-square function within the R software version 4.2.2 (*p* < 0.05).

## 3 Results

### 3.1 Seroprevalence at the herd level vs. individual level

In the face of 439 herds to be sampled (227 and 212 sheep and goat respectively), a total of 446 herds (294 and 152 sheep and goat respectively) in nine provinces were finally included in this study (Table 1).

**Table 1:** Proportion of sheep and goat herds for province.

Province	% of sheep herds	% of goat herds
Agrigento	11.2	7.5
Caltanissetta	5.7	6.5
Catania	6.9	6.6
Enna	13.5	1.2
Messina	19	38
Palermo	24.5	25.8
Ragusa	7.3	2.1
Siracusa	4.3	7.0
Trapani	7.4	4.0
	100	100

The minimum sample size for both sheep and goat herds was achieved in six provinces: Agrigento, Caltanissetta, Enna, Messina, Trapani and Ragusa. In the remaining three provinces (Palermo, Siracusa, and Catania), the minimum sample size was not achieved, with sample coverage ranging from 10 to 78.2%.

The overall mean of the regional AP in sheep herds was 71.8% (95% CI: 53.2–90.4%) (Table 2) with a highest value (100%) in Ragusa (12 herds) and Siracusa (just one sampled herd) provinces, while lower prevalence was found in Trapani (19 herds) and Messina (78 herds) with AP of 26.3 and 46.2%, respectively. Statistical differences between the provinces were found (*p* < 0.001).

**Table 2:** Overall apparent (AP) and True prevalence (TP) of MAP at herd and animal level in sheep, considered by provinces.

Species	Province	Sampled farms	AP herd-level %	Sampled heads	AP animal-level %	TP IDEXX Se = 34.9% Sp = 97.3%	TP Nielsen & Toft Se = 37% Sp = 98.5%
Sheep	Agrigento	33	75.8 (58.98–87.17)	4,356	6.6 (5.89–7.36)	12.1 (9.9–14.5)	14.4 (12.4–16.5)
	Caltanissetta	37	83.8 (68.86–92.35)	4,424	7.6 (6.85–8.40)	15.2 (12.9–17.7)	17.2 (15.07–19.50)
	Catania	10	80.0 (49.02–94.33)	1,324	9.4 (7.91–11.05)	20.8 (16.19–25.95)	22.2 (18.06–26.91)
	Enna	61	73.8 (61.56–83.16)	7,180	3.9 (3.48–4.37)	3.7 (2.41–5.19)	6.8 (5.57–8.09)
	Messina	78	46.2 (35.53–57.14)	9,883	2.3 (2.02–2.60)	0.0	2.2 (1.46–3.13)
	Palermo	43	60.5 (45.58–73.63)	2,915	2.4 (1.91–3.02)	0.0	2.5 (1.14–4.29)
	Ragusa	12	100.0 (75.75–1.00)	3,764	5.9 (5.19–6.70)	9.9 (7.73–12.41)	12.4 (10.39–14.64)
	Trapani	19	26.3 (11.81–48.79)	1,539	0.8 (0.45–1.36)	0.0	0.0
	Siracusa	1	100	278	1.4 (0.56–3.64)	0.0	0.0
		**294**	**71.8 95% CI (53.24–90.40%)**	**34,086**	**4.5 95% CI (2.1–6.8%)**	**6.8 95% CI (0.8–12.9%)**	**8.6 95% CI (2.3–14.9%)**

Significance (*p* ≤ 0.05) of χ^2^ test between provinces.

The overall mean of the AP in goat herds was 60.8% (95% CI: 37.5–84.1%) (Table 3) with a highest prevalence of reported in Siracusa (100%; 5 herds), Caltanissetta (84.2%; 19 herds), Ragusa (83.3%; 6 herds) and Catania (75%; 8 herds) provinces. In contrast the lowest AP was found in Agrigento province (39.1%, 23 herds). Statistical difference between the provinces was found (*p* < 0.05).

**Table 3:** Overall apparent (AP) and True prevalence (TP) of MAP at herd and animal level in goats, considered by provinces.

Species	Province	Sampled farms	AP herd-level %	Sampled heads	AP animal-level %	TP IDEXX Se = 34.9% Sp = 97.3%	TP Nielsen & Toft Se = 37% Sp = 98.5%
Goat	Agrigento	23	39.1 (22.16–59.21)	819	6.6 (5.09–8.50)	12.1 (7.42–18.03)	14.4 (10.11–19.73)
	Caltanissetta	19	84.2 (62.43–94.48)	1,768	5.8 (4.78–6.96)	9.6 (6.44–13.21)	12.1 (9.23–15.37)
	Catania	8	75.0 (40.93–92.85)	493	6.5 (4.64–9.02)	11.8 (6.01–19.63)	14.1 (8.83–21.18)
	Enna	13	69.2 (42.37–87.32)	657	7.8 (5.95–10.06)	15.8 (10.10–22.87)	17.7 (12.54–24.12)
	Messina	67	52.2 (40.49–63.75)	7,325	1.7 (1.42–2.01)	0	0.5 (−0.22–1.45)
	Palermo	9	44.4 (18.88–73.33)	348	4.3 (2.63–6.99)	5.0 (0–13.32)	7.9 (3.18–15.46)
	Ragusa	6	83.3 (43.65–96.99)	948	6.8 (5.32–8.53)	12.7 (8.14–18.10)	14.9 (10.77–19.80)
	Trapani	2	0.0	20	5.0 (0.89–23.61)	7.1 (−7.59–64.95)	9.86 (−3.5–62.29)
	Siracusa	5	100.0 (56.55–1.00)	602	1.5 (0.79–2.82)	0	0
		**152**	**60.8 95% CI (37.5–84.1%)**	**12,980**	**5.1 95% CI (3.4–6.8%)**	**8.2 95% CI (3.9–12.6%)**	**10.2 95% CI (5.3–15.0%)**

Significance (*p* ≤ 0.05) of χ^2^ test between provinces.

At animal-level we found 1,577 positive sheep with an overall mean of the AP of 4.5% (95% CI: 2.1–6.8%). The TP were 6.8% (95% CI: 0.8–12.9%) and 8.6% (95% CI: 2.3–14.9%) according to IDEEX and Nielsen & Toft, respectively (Table 2). The highest AP (9.4%) was found in Catania province (TP reported was 20.8 and 22.2% according to IDEXX and Nielsen & Toft values, respectively). The lowest values were found in Trapani and Siracusa provinces, with AP of 0.8 and 1.4%, respectively (Table 2). The PPV and NPV were 0.42 and 0.96, respectively. Statistical differences between the provinces were found (*p* < 0.001).

The overall mean of the AP at animal-level resulted 5.1% (95% CI: 3.4–6.8%) in 12,980 goats with TP of 8.2 (95% CI: 3.9–12.6%) and 10.2% (95% CI: 5.3–15.0%) according IDEXX and Nielsen & Toft values, respectively. The highest AP was found in Enna provinces (7.8%; 657 animals) with a resulted higher TP of 15.8 and 17.7% according to IDEEX and Nielsen & Toft values, respectively. Lowest AP were found in Siracusa (602 animals) and Messina (7,325 animals) with 1.5 and 1.7%, respectively. TP resulted nearly 0% in both cases. The PPV and NPV were 0.32 and 0.99, respectively.

### 3.2 Seroprevalence within breeds

Regarding the prevalence study within the sheep breeds, at herd-level the AP ranged from 47.9% in Pinzirita (2,560 animals) to 93.7% in crossbreed (18,848 animals), while at animal-level the AP ranged from 2.7% in Pinzirita to 5.7% in Comisana (3,914 animals) (Table 4). The TP at animal-level showed the higher values in Comisana (9.3 and 11.8% according to IDEEX and Nielsen & Toft, respectively) and Valle del Belice (6.5 and 9.3% according to IDEEX and Nielsen & Toft values, respectively). Lowest values of the TP were found in Pinzirita (0 and 3.4% according to IDEEX and Nielsen & Toft, respectively) and crossbreed (2.2 and 5.3% according to IDEEX and Nielsen & Toft, respectively). Worthy of mention, the highest AP of 18.7% found in Sarda breed (995 animals; 2 herds), with TP values slightly less than 50% according both IDEEX and Nielsen & Toft (data no show in table). Regarding the AP at herd-level within the goat breeds, we found lower overall seroprevalence than sheep breeds, with values between of 16.7 and 67.7% in Girgentana (324 animals) and Messinese (1,088 animals), respectively. Conversely, the AP at animal-level within goat breeds showed a wider range of data than sheep breeds, ranged from 0.6% in Girgentana to 8.8% in Argentata dell’Etna (1,777 heads). The TP obtained was very high in Argentata dell’Etna according both IDEEX (18.9%) and Nielsen & Toft (20.5%) values. High TP values was also found in Maltese (9.9 and 12.3% according to IDEEX and Nielsen & Toft values respectively). Conversely, TP of 0% was found in Girgentana and Derivata di Siria (453 animals) (Table 5).

**Table 4:** Overall apparent (AP) and True prevalence (TP) of MAP at herds and animal level in sheep, considered by breeds.

Species	Breeds	Sampled farms	AP herd-level	Sampled heads	AP animal-level	TP IDEXX Se = 34.9% Sp = 97.3%	TP Nielsen & Toft Se = 37% Sp = 98.5%
Sheep	Comisana	41	53.7 (38.75–67.94)	3,914	5.7 (5.01–6.47)	9.3 (7.19–11.7)	11.8 (9.9–13.99)
	Pinzirita	48	47.9 (34.47–61.67)	2,560	2.7 (2.14–3.40)	0	3.4 (1.79–5.34)
	Valle Del Belice	106	50.0 (40.65–59.35)	8,201	4.8 (4.36–5.29)	6.5 (5.16–8.04)	9.3 (8.06–10.67)
	Crossbreed	93	93.5 (86.63–97.01)	18.848	3.4 (3.15–3.67)	2.2 (1.40–3.01)	5.3 (4.65–6.11)

Significance (*p* ≤ 0.05) of χ^2^ test between sheep breeds.

**Table 5:** Overall apparent (AP) and True prevalence (TP) of MAP at herd and animal level in goats, considered by breeds.

Species	Breeds	Sampled farms	AP herd-level	Sampled heads	AP animal-level	TP IDEXX Se = 34.9% Sp = 97.3%	TP Nielsen & Toft Se = 37% Sp = 98.5%
Goat	Derivata di Siria	32	65.6 (48.31–79.59)	453	0.9 (0.34–2.25)	0	0
	Girgentana	12	16.7 (4.7–44.8)	324	0.6 (0.17–2.22)	0	0
	Maltese	9	44.4 (18.88–73.33)	645	5.9 (4.32–7.98)	9.9 (5.04–16.41)	12.3 (7.95–18.26)
	Messinese	31	67.7 (50.14–81.43)	1,088	3.5 (2.56–4.76)	2.4 (−0.45–6.39)	5.6 (2.97–9.18)
	Argentata dell’Etna	5	40.0 (11.76–76.93)	1,777	8.8 (7.55–10.19)	18.9 (15.06–23.25)	20.5 (17.04–24.47)
	Crossbreed	63	61.9 (49.56–72.88)	7,746	1.9 (1.62–2.23)	0	1.1 (0.33–2.05)

Significance (*p* ≤ 0.05) of χ^2^ test between goats breeds.

## 4 Discussion

Our study provides the first large-scale overview on MAP infection in small ruminant throughout Sicily, estimating the herd-level, animal-level and within breeds seroprevalence. Our investigation revealed a high herd-level AP of 71.8% in sheep and 60.8% in goat herds with values ranking from 26.3 to 100% and 39.1 to 84.2% depending on the province, in sheep and goat herds, respectively.

Regarding the animal-level prevalence, we found a regional AP of 4.5% in sheep with the highest values of 9.4% reported in Catania province. Overall AP within-herd in goats was 5.1%, highest value of 7.8% in Enna province was found. According to IDEEX and Nielsen & Toft values respectively, TP ranged from 6.8 to 8.6% in sheep (Table 2) and between 8.2 and 10.2 in goats (Table 3).

To date, preliminary studies have already investigated the presence of MAP infection in sheep herds in Sicily, reporting a seroprevalence of 18% in Palermo (Guercio et al., personal communication) and 3.4% in Trapani (36) provinces. Our results show higher prevalence in both provinces (60.5 and 26.3% in Palermo and Trapani respectively) proving that MAP infection was previously underestimated in these provinces and probably in the rest of the region. In Italy, although there are no large-scale surveys on the spread of MAP infection in small ruminants, a significative study carried out in Apulia (a region sited in Southern Italy) revealed a herd-level AP of 60.5%, with 3% at sheep-level and 14.5% at goat-level (16). Another study performed on dairy sheep in Marche region (central Italy) showed a higher prevalence of 73.7% at flock-level with 6.29% within-herd (37).

Concerning the MAP infection in goats, our results overlap other studies carried out recently in four Northern Italy concerning 33 dairy herds, reporting a seroprevalence of 58% at herd-level with 7.4% at animal level (38).

Regarding other livestock species, a serological study in dairy cows carried out in two neighboring Northern Italian regions (Lombardy and Veneto) that account for over 50% of the Italian dairy cattle population reported a herd-level apparent prevalence of 48 and 65%, respectively (39). Similar results was obtained in a large-scale survey in water buffaloes (*Bubalus bubalis*) in Campania reporting an apparent prevalence of infection of 54.7% (40). Our results support previous studies and confirm the high prevalence of MAP infection in Italian livestock.

Concerning the global epidemiological situation of MAP infection in small ruminants, the number of prevalence studies is low and as they differ in study design and diagnostic tests used. Consequentially, MAP prevalence in small ruminants is still unknown in many countries and the prevalence appears to be underestimated (41). According to a global survey involving 48 countries, limited to countries with available data, the estimated herd-level prevalence of MAP was higher than 10% in 5 of 11 countries for sheep and 7 of 12 countries for goats (42). The same study reported estimated seroprevalence data at animal level up to 5% in four countries both in sheep and goats and values higher of 15% in two and three countries in sheep and goats, respectively (42). Finally, Seroprevalence of MAP by ELISA in sheep and goats in different European countries was summarized by Jiménez-Martín et al. recently (43).

Regarding the hypothesis that some small ruminant breeds are more resistant to MAP infection than others, experimental data are very limited and evidence of experimental infection on different breeds is lacking. Several studies report that some sheep breeds may develop clinical signs of PTB rather than others (44–46). Other studies showed that genetics may play a role in the susceptibility to PTB in sheep and goats (31, 47). Within the small ruminant breeds tested in study, we found AP values at animal-level between 2.7 (Pinzirita) and 5.7% (Comisana) in sheep and 0.6% (Girgentana) and 8.8% (Argentata dell’Etna) in goats. Notable of TP differences between the breeds stand out, with values between 0 (Pinzirita) and 11.8% (Comisana) in sheep and 0 (Derivata di Siria and Girgentana) and 20.5% (Argentata dell’Etna), suggesting the hypotetical implication the of genetic factors in the predisposition to MAP infection. In addition, the high seroprevalence values at animal-level (AP 18.7%; TP ~50%) in the two Sarda herds included in the study do not allow an analysis of statistical significance, but suggest the need for further investigations on this breed that appears to be the most bred in Italy.

The breeding of native breeds in Sicily represents an important source of income for the livestock sector especially to produce typical dairy products derived from raw milk which support people in rural where a semi-extensive farming is in place and PTB is rarely diagnosed.

Considered a similar breeding system that exposes all animals to several risk factors for PTB infection in Sicily, our findings strengthen the hypothesis that genetic factors within breed may determine susceptibility/resistance to MAP infection and highlight the importance of the preservation of native breeds as a reservoir of natural resistance against some infectious diseases. In this regard, genetic resistance/susceptibility to infectious diseases, in particular to Scrapie and Maedi-Visna virus (48–50) in Sicilian small ruminant breeds were already reported.

According to specific criteria of the European Union Animal Health Law – Regulation (UE) 2016/429 (AHL), PTB was included under Category E (listed disease for which there is a need for surveillance within the Union, of which Article 9(1)(e) for the listed animal species Bison spp., Bos spp., Bubalus spp., Ovis spp., Capra spp., Camelidae and Cervidae. Inclusion in category E entails the obligation of surveillance as well as notification of the disease to the competent authorities. Other categories were disregarded, mainly due to the low individual sensitivity of diagnostic tests currently in use, together with the difficulties of declaring countries, areas and herds paratuberculosis-free officially (42). To date, MAP infection prevalence in ruminants is considered underestimated worldwide. In Italy, the Ministry of Health, in application of the AHL, recently has approved the adoption on the national territory of “*Guidelines for the surveillance, the adoption of control plans and the assignment of health qualification to establishments of sensitive species (Cattle, Buffalo, Sheep, Goats) against paratuberculosis”* (GU General Series n.10 of 13-01-2023). These guidelines originate from the need for surveillance of the PTB throughout the national territory, in view of recognized endemicity in Italian herds and the awareness of having data that underestimate both its prevalence and incidence, these, essential for effective surveillance; its application, has as general objective of providing the right indications to implement surveillance on the national territory (definition of the roles, tasks and responsibilities of public and private veterinarians and operators; definition of “suspected” and “confirmed” cases and on the rules of reporting and notification of PTB). Despite the profound socio-economic impact of PTB in small ruminants, few studies have been published worldwide and consequently prevention and control programs have not been established in many countries.

Unfortunately, large-scale studies on the seroprevalence of PTB still presuppose limitations that we have also encountered. The sample sizes per herd in our study varied considerably, thus the TP was estimated only at animal-level and not at herd-level. In addition, the low sensitivity of the available diagnostic tests hampers the precision of diagnosis in subclinical infection and/or in early stage of disease with consequent underreported outbreaks.

Our study represents the first large-scale survey on the prevalence of PTB in small ruminants in Sicily, according to the Italian Ministry of Health guidelines for surveillance of the disease throughout the national territory. The data obtained showed a high prevalence of the disease in Sicilian herds which varies according to the species, breed, and province, highlighting the need to implementing specific PTB control plans in small ruminants.

This study represents the first step to better understand the epidemiology of the disease in semi-extensive breeding contexts, typical of Mediterranean basin, suggesting the need for further investigations to evaluate all the risk factors in the herds, included the presence of candidate genes for resistance/susceptibility to PTB in native sheep and goat breeds to helping the implementation of control plans in the near future.

## Data availability statement

The raw data supporting the conclusions of this article will be made available by the authors, without undue reservation.

## Ethics statement

Ethical approval was not required for the study involving animals in accordance with the local legislation and institutional requirements because this study did not involve controls under EU Directive 2010 (2010/63/EU) and blood collection was not required with the benefit of animal welfare. The large-scale study on MAP seroprevalence in small ruminant farms in Sicily was carried out on sera samples provided by the annual brucellosis monitoring plan in force in Sicily, collected and archived during the three years in study.

## Author contributions

VD: Conceptualization, Formal analysis, Project administration, Supervision, Writing – review & editing. DI: Data curation, Writing – review & editing. SM: Data curation, Formal analysis, Writing – original draft, Writing – review & editing. MT: Data curation, Formal analysis, Writing – review & editing. SM: Conceptualization, Investigation, Writing – review & editing. AMFM: Investigation, Writing – review & editing. BA: Investigation, Writing – review & editing. RC: Investigation, Writing – review & editing. MV: Formal analysis, Writing – review & editing. DV: Investigation, Writing – review & editing. FPC: Investigation, Writing – review & editing. MF: Conceptualization, Formal analysis, Supervision, Writing – review & editing.

## References

[1] Roller M Hansen S Knauf-Witzens T Oelemann WMR Czerny C-P Abd El Wahed A. *Mycobacterium avium* subspecies paratuberculosis infection in zoo animals: a review of susceptibility and disease process. Front Vet Sci. (2020) 7:572724. doi: 10.3389/fvets.2020.572724, PMID: 33426014 PMC7785982

[2] Garvey M. *Mycobacterium avium* subspecies paratuberculosis: a possible causative agent in human morbidity and risk to public health safety. Open Vet J. (2018) 8:172–81. doi: 10.4314/ovj.v8i2.10, PMID: 29911021 PMC5987349

[3] Feller M Huwiler K Stephan R Altpeter E Shang A Furrer H. *Mycobacterium avium* subspecies paratuberculosis and Crohn’s disease: a systematic review and meta-analysis. Lancet Infect Dis. (2007) 7:607–13. doi: 10.1016/S1473-3099(07)70211-6, PMID: 17714674

[4] Sechi LA Dow CT. *Mycobacterium avium* ss. Paratuberculosis zoonosis – the hundred year war– beyond Crohn’s disease. Front Immunol. (2015) 6:96. doi: 10.3389/fimmu.2015.00096, PMID: 25788897 PMC4349160

[5] Cossu A Rosu V Paccagnini D Cossu D Pacifico A Sechi LA. MAP 3738c and Mpt D are specific tags of *Mycobacterium avium* subsp. paratuberculosis infection in type I diabetes mellitus. Clin Immunol. (2011) 141:49–57. doi: 10.1016/j.clim.2011.05.002, PMID: 21664191

[6] Frau J Coghe G Lorefice L Fenu G Cocco E. Infections and multiple sclerosis: from the world to Sardinia, from Sardinia to the world. Front Immunol. (2021) 12:728677. doi: 10.3389/fimmu.2021.728677, PMID: 34691035 PMC8527089

[7] Dow CT. M. Paratuberculosis and Parkinson’s disease – is this a trigger. Med Hypotheses. (2014) 83:709–12. doi: 10.1016/j.mehy.2014.09.025, PMID: 25459140

[8] Dow CT Ellingson JLE. Detection of *Mycobacterium avium* ss. paratuberculosis in Blau syndrome tissues. Autoimmune Dis. (2010) 2010:1–5. doi: 10.4061/2010/127692, PMID: 21152214 PMC2989750

[9] D’Amore M Lisi S Sisto M Cucci L Dow CT. Molecular identification of *Mycobacterium avium* subspecies paratuberculosis in an Italian patient with Hashimoto’s thyroiditis and Melkersson–Rosenthal syndrome. J Med Microbiol. (2010) 59:137–9. doi: 10.1099/jmm.0.013474-0 19797462

[10] Aboagye G Rowe MT. Occurrence of *Mycobacterium avium* subsp. paratuberculosis in raw water and water treatment operations for the production of potable water. Water Res. (2011) 45:3271–8. doi: 10.1016/j.watres.2011.03.029, PMID: 21529886

[11] Gill CO Saucier L Meadus WJ. *Mycobacterium avium* subsp. paratuberculosis in dairy products, meat, and drinking water. J Food Prot. (2011) 74:480–99. doi: 10.4315/0362-028X.JFP-10-301, PMID: 21375889

[12] Kuenstner L Kuenstner JT. *Mycobacterium avium* ssp. paratuberculosis in the food supply: a public health issue. Front. Public Health. (2021) 9:647448. doi: 10.3389/fpubh.2021.647448, PMID: 34336758 PMC8319643

[13] Windsor PA. Paratuberculosis in sheep and goats. Vet Microbiol. (2015) 181:161–9. doi: 10.1016/j.vetmic.2015.07.019 26255556

[14] Stonos N Bauman C Menzies P Wootton SK Karrow NA. Prevalence of small ruminant lentivirus and *Mycobacterium avium* subsp. paratuberculosis co-infection in Ontario dairy sheep and dairy goats. Can J Vet Res. (2017) 81:155–9. PMID: 28408784 PMC5370542

[15] Idris SM Eltom KH Okuni JB Ojok L Elmagzoub WA El Wahed AA. Paratuberculosis: the hidden killer of small ruminants. Animals. (2021) 12:12. doi: 10.3390/ani12010012, PMID: 35011118 PMC8749836

[16] Sardaro R Pieragostini E Rubino G Petazzi F. Impact of *Mycobacterium avium* subspecies paratuberculosis on profit efficiency in semi-extensive dairy sheep and goat farms of Apulia, southern Italy. Prev Vet Med. (2017) 136:56–64. doi: 10.1016/j.prevetmed.2016.11.013, PMID: 28010908

[17] Stau A Seelig B Walter D Schroeder C Ganter M. Seroprevalence of *Mycobacterium avium* subsp. paratuberculosis in small ruminants in Germany. Small Rumin Res. (2012) 105:361–5. doi: 10.1016/j.smallrumres.2012.03.008

[18] Mercier P Baudry C Beaudeau F Seegers H Malher X. Estimated prevalence of *Mycobacterium avium* subspecies *paratuberculosis* infection in herds of dairy goats in France. Vet Rec. (2010) 167:412–5. doi: 10.1136/vr.c4454, PMID: 20834001

[19] Michel A. Paratuberculosis in sheep: an emerging disease in South Africa. Vet Microbiol. (2000) 77:299–307. doi: 10.1016/S0378-1135(00)00315-1, PMID: 11118715

[20] Zhao L Wang Y Wang J-L Zhao W-H Cheng H-X Ma Y-M. Serological investigation and genotyping of *Mycobacterium avium* subsp. paratuberculosis in sheep and goats in Inner Mongolia, China. PLoS ONE. (2021) 16:e0256628. doi: 10.1371/journal.pone.0256628, PMID: 34492040 PMC8423245

[21] Debien E Hélie P Buczinski S Lebœuf A Bélanger D Drolet R. Proportional mortality: a study of 152 goats submitted for necropsy from 13 goat herds in Quebec, with a special focus on caseous lymphadenitis. Can Vet J. (2013) 54:581–7. PMID: 24155449 PMC3659454

[22] Pithua P Kollias NS. Estimated prevalence of caprine paratuberculosis in Boer goat herds in Missouri, USA. Vet Med Int. (2012) 2012:1–5. doi: 10.1155/2012/674085, PMID: 23251834 PMC3515964

[23] Freitas TD Azevedo SSD Silva MLCR Garino Júnior F Santos CDSAB Clementino I. Epidemiological characterization and risk factors associated with *Mycobacterium avium* subsp. paratuberculosis infection in dairy goats in the Brazilian semiarid region. Semin Cienc Agrar. (2015) 36:267. doi: 10.5433/1679-0359.2015v36n1p267

[24] Eamens GJ Marsh IM Plain KM Whittington RJ. Paratuberculosis (Johne’s disease) In: Australian and New Zealand standard diagnostic procedures. Canberra, Australia: Department of Agriculture, water and the environment, Australian government (2015). 1–68.

[25] Elsohaby I Fayez M Alkafafy M Refaat M Al-Marri T Alaql FA. Serological and molecular characterization of *Mycobacterium avium* subsp. paratuberculosis (MAP) from sheep, goats, cattle and camels in the Eastern Province, Saudi Arabia. Animals. (2021) 11:323. doi: 10.3390/ani11020323, PMID: 33525431 PMC7911684

[26] Hailat NQ Hananeh W Metekia AS Stabel JR Al-Majali A Lafi S. Pathology of subclinical paratuberculosis (Johne’s disease) in Awassi sheep with reference to its occurrence in Jordan. Vet Med. (2010) 55:590–602. doi: 10.17221/2947-VETMED

[27] Selim A Abdelhady A Abdelrahman A. Ovine Paratuberculosis: Seroprevalence and comparison of fecal culture and direct fecal PCR assay. Comp Immunol Microbiol Infect Dis. (2021) 74:101526. doi: 10.1016/j.cimid.2020.101526, PMID: 32861473

[28] Idris SM Ali EA Elmagzoub WA Okuni JB Mukhtar ME Ojok L. First report on ovine paratuberculosis in the Sudan: diagnosis using different techniques. Animals. (2022) 12:3312. doi: 10.3390/ani12233312, PMID: 36496833 PMC9737915

[29] Benazzi S Hamidi M Schliesser T. Paratuberculosis in sheep flocks in Morocco: a serological, microscopical and cultural survey. J Veterinary Med Ser B. (1996) 43:213–9. doi: 10.1111/j.1439-0450.1996.tb00308.x 8767767

[30] Vitale M Migliore S La Giglia M Alberti P Di Marco Lo Presti V Langeveld JPM. Scrapie incidence and PRNP polymorphisms: rare small ruminant breeds of Sicily with TSE protecting genetic reservoirs. BMC Vet Res. (2016) 12:141. doi: 10.1186/s12917-016-0766-9, PMID: 27417309 PMC4946234

[31] Cecchi F Russo C Iamartino D Galiero A Turchi B Fratini F. Identification of candidate genes for paratuberculosis resistance in the native Italian Garfagnina goat breed. Trop Anim Health Prod. (2017) 49:1135–42. doi: 10.1007/s11250-017-1306-8, PMID: 28526988

[32] Della Croce G Forletta R Ricca R. Indagine dellla paratubercolosi dei piccoli ruminanti: incidenza della malattia nelle provincie di Caltaniessetta e Agrigento. Atti SISVET, 927–992 Presented at the congress of Società italiana delle scienze veterinarie (SISVET) Riccione (1993).

[33] Arrigoni M. Taddei R. (2008) *Mycobacterium avium*s subsp. paratuberculosis: caratteristiche biologiche e genotipiche. Convegno sulla diagnostica di laboratorio in sanità animale, Roma, 29 Maggio 2008: 1–8.

[34] Rogan W. J. Gladen B. Estimating prevalence from the results of a screening test. Am J Epidemiol. (1978) 107:71–6. doi: 10.1093/oxfordjournals.aje.a112510 623091

[35] Brown LD Cat TT Das Gupta A. Interval estimation for a proportion. Stat Sci. (2001) 16:101–33. doi: 10.1214/ss/1009213286

[36] Villari S. Castiglione F. Monteverde V., “Seroprevalence of *Mycobacterium avium* subsp. paratuberculosis (MAP) in ovine and caprine farms in Trapani, Sicily,” in Proceedings of the 17th international congress of Mediterranean Federation of Health and Production of ruminants, pp. 117–118, Perugia, Italy (2009).

[37] Anna Rita A Victor NN Silvia P Luciana P Anastasia D Vincenzo C. Ovine paratuberculosis: a seroprevalence study in dairy flocks reared in the Marche region, Italy. Vet Med Int. (2011) 2011:1–10. doi: 10.4061/2011/782875, PMID: 21876850 PMC3163026

[38] Gaffuri A Barsi F Magni E Bergagna S Dellamaria D Ricchi M. Paratuberculosis, animal welfare and biosecurity: a survey in 33 northern Italy dairy goat farms. Animals. (2023) 13:2346. doi: 10.3390/ani13142346, PMID: 37508122 PMC10376716

[39] Pozzato N Capello K Comin A Toft N Nielsen SS Vicenzoni G. Prevalence of paratuberculosis infection in dairy cattle in northern Italy. Prev Vet Med. (2011) 102:83–6. doi: 10.1016/j.prevetmed.2011.07.001, PMID: 21807432

[40] Martucciello A Galletti G Pesce A Russo M Sannino E Arrigoni N. Short communication: seroprevalence of paratuberculosis in Italian water buffaloes (*Bubalus bubalis*) in the region of Campania. J Dairy Sci. (2021) 104:6194–9. doi: 10.3168/jds.2020-19022, PMID: 33685689

[41] Nielsen SS Toft N. A review of prevalences of paratuberculosis in farmed animals in Europe. Prev Vet Med. (2009) 88:1–14. doi: 10.1016/j.prevetmed.2008.07.003, PMID: 18817995

[42] Whittington R Donat K Weber MF Kelton D Nielsen SS Eisenberg S. Control of paratuberculosis: who, why and how. A review of 48 countries. BMC Vet Res. (2019) 15:198. doi: 10.1186/s12917-019-1943-4, PMID: 31196162 PMC6567393

[43] Jiménez-Martín D García-Bocanegra I Risalde MA Fernández-Molera V Jiménez-Ruiz S Isla J. Epidemiology of paratuberculosis in sheep and goats in southern Spain. Prev Vet Med. (2022) 202:105637. doi: 10.1016/j.prevetmed.2022.105637, PMID: 35378433

[44] Delgado L Marín JFG Muñoz M Benavides J Juste RA García-Pariente C. Pathological findings in young and adult sheep following experimental infection with 2 different doses of *Mycobacterium avium* subspecies paratuberculosis. Vet Pathol. (2013) 50:857–66. doi: 10.1177/0300985813476066, PMID: 23390077

[45] Lugton I. Cross-sectional study of risk factors for the clinical expression of ovine Johne’s disease on New South Wales farms. Aust Vet J. (2004) 82:355–65. doi: 10.1111/j.1751-0813.2004.tb11104.x, PMID: 15267095

[46] Smeed JA Watkins CA Rhind SM Hopkins J. Differential cytokine gene expression profiles in the three pathological forms of sheep paratuberculosis. BMC Vet Res. (2007) 3:18. doi: 10.1186/1746-6148-3-18, PMID: 17697353 PMC1994670

[47] Reddacliff LA Beh K McGregor H Whittington RJ. A preliminary study of possible genetic influences on the susceptibility of sheep to Johne’s disease. Aust Vet J. (2005) 83:435–41. doi: 10.1111/j.1751-0813.2005.tb13087.x, PMID: 16035186

[48] Migliore S Agnello S Chiappini B Vaccari G Mignacca SA di Marco Lo Presti V. Biodiversity and selection for scrapie resistance in goats: genetic polymorphism in “Girgentana” breed in Sicily, Italy. Small Rumin Res. (2015) 125:137–41. doi: 10.1016/j.smallrumres.2015.01.029

[49] Migliore S Agnello S D’Avola S Goldmann W di Marco Lo Presti V Vitale M. A cross-sectional study of PRNP gene in two native Sicilian goat populations in Italy: a relation between prion gene polymorphisms and scrapie incidence. J Genet. (2017) 96:319–25. doi: 10.1007/s12041-017-0776-9, PMID: 28674232

[50] Tumino S Tolone M Galluzzo P Migliore S Sechi T Bordonaro S. Alternative molecular tools for the fight against infectious diseases of small ruminants: native Sicilian sheep breeds and Maedi-Visna genetic susceptibility. Animals (Basel). (2022) 12:1630. doi: 10.3390/ani12131630, PMID: 35804527 PMC9264923

